# Promoting positive physical activity behaviors for children and adolescents undergoing acute cancer treatment: Development of the CanMOVE intervention using the Behavior Change Wheel

**DOI:** 10.3389/fped.2022.980890

**Published:** 2022-10-13

**Authors:** Sarah L. Grimshaw, Nicholas F. Taylor, Rachel Conyers, Nora Shields

**Affiliations:** ^1^School of Allied Health, Human Services and Sport, La Trobe University, Melbourne, VIC, Australia; ^2^Murdoch Children’s Research Institute, Melbourne, VIC, Australia

**Keywords:** cancer, child, adolescent, physical activity, Behavior Change Wheel, complex intervention development

## Abstract

**Background:**

Increasing participation in physical activity has the potential to improve outcomes for children and adolescents with cancer during treatment and into survivorship. The aim of this study is to outline the theoretical process behind development of CanMOVE, a behavior change intervention designed to increase physical activity for children and adolescents with cancer.

**Study design:**

This study followed a theoretical design process consistent with the Behavior Change Wheel to inform the design of a complex intervention.

**Materials and methods:**

The three stages of the Behavior Change Wheel intervention design process include: (1) understanding physical activity behavior within the pediatric cancer setting, (2) identifying potential intervention functions, and (3) identifying appropriate behavior change and implementation strategies. Qualitative and behavior change literature relevant to the pediatric cancer treatment setting were used to inform each stage.

**Results:**

An individualized and flexible approach to physical activity promotion that considers intrinsic factors specific to the child/adolescent and their environment is required. Fifteen behavioral change strategies were identified to form the intervention components of CanMOVE. Implementation strategies were identified to build motivation, opportunity and capacity toward increasing physical activity behaviors. Key intervention components of CanMOVE include standardized assessment and monitoring (physical activity, physical function, and health-related quality of life), provision of an activity monitor to both child/adolescent and parent, and one-on-one capacity building sessions with a healthcare professional. Capacity building sessions include education, goal setting, an active supervised physical activity session, barrier identification and problem solving, and action planning.

**Conclusion:**

CanMOVE is a novel approach to physical activity promotion in the pediatric cancer treatment setting. The use of a theoretical intervention design process will aid evaluation and replication of CanMOVE when it is assessed for feasibility in a clinical setting. The design process utilized here can be used as a guide for future intervention development.

## Introduction

Childhood cancer and its treatment can cause adverse physical effects (1–5), evident from as early as one week following diagnosis (6). Muscle loss, reduced fitness, fatigue, and motor impairment are prevalent among children and adolescents undergoing acute cancer therapy. These adverse effects are not limited to the acute treatment phase. Adults who have undergone childhood cancer treatment display high levels of sedentary behavior, can experience lifelong disability and impairment, and are at an increased risk of chronic disease and premature mortality (7–9). A growing number of childhood cancer survivors are reaching adulthood, which increases the burden of these adverse outcomes (10). Intervening early could work to mitigate these negative effects and promote improved physical function and wellbeing in the immediate and long-term.

Physical activity is vital to health and development (11, 12), yet, children and adolescents undergoing acute cancer treatment are less active than their age-matched peers (13, 14). Children and adolescents can receive intensive cancer treatments over the course of many months (15). Over this time, adverse treatment effects can compromise a child’s ability to be physically active and functionally independent. For this population, physical activity has a role to play in managing treatment-related effects, preventing (or minimizing) declines in physical function and mental health, maintaining physical literacy skills and promoting active lifestyles (16, 17). Managing these negative factors through proactive physical activity promotion could help to maximize their physical function and participation during cancer treatment. This could in turn have a positive impact on long term health outcomes, such as reducing the risk of physical impairment, metabolic syndrome and cardiovascular morbidity (18, 19). There is growing evidence to support the benefits of physical activity for children with cancer (20–22), yet the barriers to physical activity in this setting are complex, and there is little consensus regarding how to implement feasible, equitable and sustainable interventions (23, 24).

Physical activity encompasses any bodily movement resulting in energy expenditure (25). As a sub-section of physical activity, literature supports the benefits of supervised exercise (26–30). Supervised exercise interventions have strong attendance and adherence rates, and numerous systematic reviews report its safety and benefits (20, 24, 31–33). However, supervised exercise often target impairments alone, and tie physical activity engagement to the presence of a trained professional. They are also costly. In treatment centers with a high volume of annual cases it can be challenging, from a funding perspective, to provide such services to all families throughout treatment. Promoting physical activity in its broadest sense, from a behavior change perspective, could help families to independently incorporate more physical activity into their daily routine (34–36). This has the potential to alleviate reliance on supervised exercise sessions alone, allowing a more nuanced and targeted approach to service delivery; whereby, more intensive support is provided to children/adolescents if, and when, it is needed.

Complex interventions comprise several interacting and flexible components, have a number of varying outcomes and involve complex behaviors (37). Physical activity is a complex behavior (38); for positive change, complex interventions that consider individual and environmental factors are required (38–40). Implementing strategies that target physical activity behavior using complex intervention design strategies are yet to be thoroughly explored in the acute pediatric cancer setting. There are examples of complex physical activity interventions within the acute cancer treatment setting (41–44), yet these examples either lack a clear theoretical underpinning or fail to incorporate strategies that target the child/adolescent and their social and physical environment.

The UK’s Medical Research Council approach to complex intervention design requires a transparent, and systematic process that articulates the theoretical basis for the intervention (45). Interventions are commonly designed without formal analysis of the behavior to be targeted, nor the theorized mechanism of action. The theoretical underpinning of a complex intervention describes how the intervention is expected to work through outlining the expected causal pathways between the intervention components, the expected outcomes and how contextual factors might influence these (46). Defining and undertaking a theoretical approach to intervention design has many benefits. It helps researchers analyze the problem, understand how an intervention can work, assess effectiveness and ultimately improves replicability and clinical implementation of results (47). Interventions designed *via* a theoretical process are considered to be more effective in leading to lasting change (48).

The Behavior Change Wheel is a framework that integrates 19 existing behavior change frameworks into one model. The components of the Behavior Change Wheel can be used to explain physical activity behavior (49), and to guide intervention design. This framework can be applied across any type of behavior and setting (50), and has been used in various health contexts to design complex physical activity interventions (51–54). The Behavior Change Wheel necessitates consideration of what internal conditions specific to the individual, and their social and physical environment need to be in place for the target behavior to be achieved (50). The COM-B component of the Behavior Change Wheel provides the method for understanding the behavior theoretically. Other theoretical frameworks such as The Transtheoretical Model of Behavior Change, Health Promoting Behavior, Theory of Planned Behavior, and Health Belief Model are commonly cited in the context of complex intervention design. These models can be helpful to predict, explain or describe behavior, yet have limitations for intervention design as they do not require in-depth analysis of the target behavior, nor link theoretical constructs to mechanisms of change (50). The Behavior Change Wheel helps researchers design interventions through linking potential intervention components with the target behavior, population and environment in which they will be delivered (48).

The Behavior Change Wheel was used here to design a complex intervention to promote positive changes in physical activity behavior specifically for children and adolescents receiving acute cancer treatment. This paper outlines the theoretical process undertaken. The decision-making process that led to the resultant intervention “CanMOVE” will be described in terms of the behavior change techniques selected and their mode of delivery. CanMOVE will subsequently be piloted for feasibility.

## Materials and methods

**FIGURE 1:**
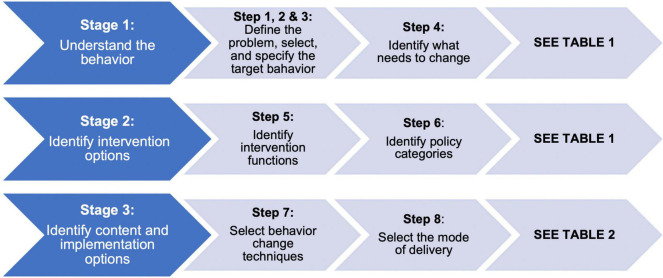
Behavior Change Wheel stages of intervention design.

The Behavior Change Wheel was the theoretical framework used to inform the design of CanMOVE (50). This intervention aimed to target school aged children (5–16 years) who were undergoing acute cancer treatment. The definition of acute cancer treatment includes hematopoietic stem cell transplantation and all treatment phases except the ‘maintenance phase’ of leukemia therapy. The research team members worked collaboratively through the three stages of this design process outlined below (Figure 1).

### Stage 1: Understand the behavior (steps 1–4)

Steps 1–3 define the problem and identify a specific behavior to change. Steps 1–3 were pre-determined prior to undergoing this design process. As described in the introduction, CanMOVE aims to proactively attenuate the negative physical health and participation restrictions observed for children with cancer. The target behavior, physical activity, was determined based on available evidence outlining that children and adolescents undergoing cancer treatment are less physically active than age-matched peers, and the potential positive effects of improved physical engagement (7, 8, 13, 14).

Step 4 analyzed what needs to change in a person, and their environment to facilitate change in the target behavior. The central components of the Behavior Change Wheel, the COM-B model, guided analysis within this step. The COM-B model proposes that for someone to undertake a particular behavior they need to be physically and psychologically *capable*, process the want or need to undertake the behavior *(motivation)*, and have the social and physical *opportunity* to engage in the behavior (50). Each of these components were evaluated on their potential contribution to physical activity behavior specifically for children and adolescents in the acute cancer setting. Data from our qualitative study were used to inform this evaluative process (55). Data were analyzed thematically, first via an inductive process to identify emergent themes, and second via a deductive process whereby the resultant themes were mapped to each of the COM-B components. Results from additional relevant qualitative literature that included insights from child and adolescent perspectives were also used (56–58). Based on the identified reasons for reduced levels of physical activity, a list of potential pathways to create change was generated.

### Stage 2: Identify intervention options (steps 5 and 6)

Stage 2 determines the types of intervention functions and policy categories that could be applied to bring about change in the target behavior. Intervention functions represent the type of intervention to be implemented and policy categories are decisions made by authorities concerning those interventions (50). Factors identified in stage 1 as contributing to physical activity behavior were mapped to potential intervention functions. This process ensures intervention techniques target the specific population and their environment. For example, skills training may be appropriate where there is a lack of skill but will be less helpful if a lack of motivation to perform the skill is the underlying reason for the behavior (48). Identifying potential policy category strategies was beyond the scope of this study.

### Stage 3: Identify content and implementation options (steps 7 and 8)

Using the intervention functions identified in stage 2, stage 3 involved selecting behavioral change techniques that could form the different components of the intervention. Behavior change techniques are the “active ingredients” selected to comprise the intervention and facilitate a change in behavior. Clear identification and definition of the behavioral change techniques selected is key to the analysis of how an intervention works; it allows the researcher to accurately describe the intervention, and aids identification of the specific techniques effective in altering behavior (59). The CALO-RE (Coventry, Aberdeen, and London – Refined) taxonomy was used to define the selected behavior change techniques as it was specifically designed to describe physical activity and healthy eating interventions (59).

For each of the selected behavior change strategies, it was then decided how they will be delivered to the target population. Selection of behavior change techniques and their delivery mode was informed through evidence-based analysis of literature relevant to physical activity in the acute cancer treatment setting and physical activity behavior change theory. It was through this decision-making process – identifying which behavior change techniques to use, and the most effective mode of delivery – that the components of CanMOVE were determined.

## Results

### Stage 1: Understand the behavior (steps 1–4)

A summary of how each of the COM-B components (capability, motivation, opportunity) contribute to physical activity behavior within the acute pediatric cancer setting can be viewed in Table 1. Results from Stage 1 highlight the diverse nature of the barriers and facilitators to physical activity that exist.

**TABLE 1:** Behavior Change Wheel stage 1 and 2.

		Stage 1		Stage 2
	
COM-B behavioral determinants		Reasons for inactivity (55–58)	What can be changed?	Intervention functions*
Capability	Psychological	Lack of knowledge: why and how to be physically active, potential benefits and risks Reduced mental capacity and stamina Mental health issues: lost confidence, embarrassment, fear, anger	Provide education regarding benefits of physical activity Practical demonstration about safe physical activity Identify mental health issues and initiate prompt referral for management services for support	Education, modeling, training, enablement
	Physical	Reduced physical ability and function: strength, balance, co-ordination, physical impairment Treatment side effects: medically unwell, fatigue, nausea, pain	Identify declines in function and initiate prompt referral to physiotherapy for assessment and treatment Identify unwanted treatment side effects and initiate prompt referral to medical team for review for management	Enablement
Motivation	Reflective	Foreign hospital environment No routine or access to independent ADLs No desire to be physically active Loss of independence and choice Lack of joy with movement Unaware of current level of physical activity (possibly reduced) Unaware of current level of function (possibly reduced)	Facilitate changes to the physical environment to promote physical activity Provide feedback on activity and sedentary behavior Provide feedback on physical function Provide support, encouragement and positive feedback Provide incentive to be physically active	Training, persuasion, incentivization, environmental restructuring
	Automatic	Negative values and beliefs toward physical activity during cancer treatment Perceived risk No perceived benefit Conflicting priorities Reduced self-efficacy toward physical activity	Education regarding potential benefits of physical activity Dispel fears regarding risks of physical activity Education and demonstration on how to safely be physically active	Education, modeling
Opportunity	Physical	Medically imposed physical restriction to movement Medical attachments Need for mobility aid use Lack of environmental cues Unstimulating environment Lack of engaging equipment Lack of space Restrictive hospital rules and policy Lack of access to previous sporting activities and environments	Facilitate changes to the physical environment to promote physical activity: access to other environments, access to toys and equipment, time detached from medical equipment, restrict sedentary activities Provision of appropriate mobility aids	Training, environmental restructuring, restriction
	Social	Lack of positive modeling and social cues Lack of social interaction Lack of availability of specialized services and staff Negative parental values and beliefs toward physical activity Reduced parental capacity – mentally and physically Values of treatment team not aligned with physical activity	Education provided to other oncology HCP about the importance of physical activity Facilitate social interactions on the ward, attendance to groups Increase social support: friends, family, staff Provide education parents and treating team about the benefits of physical activity and how to facilitate it Engage parents and treating staff in strategies to overcome physical activity	Education, training, environmental restructuring,

ALDs, activities of daily living; HCP, healthcare professional.

*Behavior Change Wheel intervention functions (50): Education, increasing knowledge or understanding. Persuasion, using communication to induce positive or negative feelings or stimulate action; Incentivization, creating expectation of reward; Coercion, creating an expectation of punishment or cost; Training, imparting skills; Restriction, using rules to reduce the opportunity to engage in the target reducing the behavior; Environmental restructuring, changing the physical or social context; Modeling, providing an example for people to aspire to or imitate; Enablement, increasing means/reducing barriers to increase capability or opportunity.

Challenges to physical activity can vary from one child to another depending on their environment, cancer type, support network, treatment regimen, emotional and physical states. In addition, barriers to physical activity can change for each individual child over the course of their acute treatment phase, which can span many months (55). A child/adolescent’s capacity to engage in physical activity can be limited by physical impairments caused by treatment side effects but also through a lack of knowledge, fear, and impaired mental health. Motivation can be impacted through spending large amounts of time in the hospital environment (both in-patient and out-patient setting), reduced physical ability, a loss of independence and freedom, and a lack of joy with movement. Opportunities to be physically active can be restricted through experiencing isolation from friends and family, residing in unstimulating environments, restricted participation in daily routines and not having access to sports equipment or toys (55–58). To address the unique characteristics of each child/adolescent and their context, multi-layered, individualized and flexible solutions are needed. Solutions need to acknowledge the heterogeneity of this population. They also need to consider the variability that exists for a child as they move through different treatment phases and have varying medical and support needs.

Many factors identified in the COM-B model are not immediately modifiable. For example, the physical layout of a ward or day oncology unit, the necessity of medical treatments, intravenous lines, resource availability, infection risks and hospital policies. In identifying potential pathways to create behavior change, focus was given to identifying ways to maximize physical activity within these constraints.

### Stage 2: Identify intervention options (steps 5 and 6)

A summary of the identified intervention functions can be viewed in Table 1. Education, modeling, training, enablement, providing incentives and environmental restructuring (50) were identified as approaches that could affect physical activity behavior.

### Stage 3: Identify content and implementation options (steps 7 and 8)

Based on stage 1 and 2, 15 behavioral change strategies to implement within CanMOVE were identified. Table 2 outlines CanMOVE’s intervention components, how they will be delivered, and the behavior change techniques selected. Also depicted are how each component is linked to the previously identified intervention functions.

**TABLE 2:** Behavior Change Wheel stage 3.

Intervention component	Mode of delivery	COM-B behavioral determinant	Intervention function*	CALO-RE BCT (59)

*Phase 1*				
*Assessment, monitoring and feedback*				
Standardized assessment of physical function, physical activity and HRQOL	Complete standardized assessments at two time points	Motivation	Education, persuasion	Provide feedback on performance
Provide feedback on performance on standardized assessments	One-on-one session with participant/parent and trial CanMOVE HCP	Motivation	Persuasion	Provide feedback on performance
Refer to specialized services as indicated	± Referral to services			Plan social support

***Phase 2***				
***Capacity building: Theme 1***				
***“Let’s find a reason for you to be physically active”***				

Provide written and verbal education regarding physical activity definition, benefits, evidence-based standards and recommendations	One-on-one session with participant/parent and CanMOVE HCP	Capability, motivation	Education, training, persuasion	Provide information about health consequences of behavior in general Provide information about health consequences of behavior to the individual
Set a steps per day goal	Provide activity monitor to participant: set steps/day target on device and visually display goal in environment	Motivation	Persuasion, incentivization	Goal setting (behavior) Environmental restructure Teach to use prompts
Provide capability to self-monitor steps/day	Child/adolescent to wear activity monitor at wrist Set prompts on activity monitor to indicate progress toward goal (50, 75, and 100% celebrations)	Motivation	Persuasion, incentivization	Prompt self-monitoring of behavior Provide feedback on performance
Engage family in goal attainment	Provide activity monitor to parent to wear and work together with child/adolescent to achieve steps/day goal	Motivation	Persuasion	Plan social support
Engage treating team in goal attainment	Visually display steps/day goal in environment Record steps/days goal in medical record	Motivation	Persuasion	Plan social support

***Capacity building: Theme 2***				
***“Let’s explore how you can be more physically active”***				

Brainstorming of possible ways to be physically active in current setting	One-on-one session with participant/parent and CanMOVE HCP	Capability, motivation	Enablement	Provide information on where and when to perform the behavior
Supervised participation in physical activity		Capability, motivation, opportunity	Training, enablement	Model/demonstrate the behavior

***Capacity building: Theme 3***				
***“Let’s make a physical activity plan”***				

Review steps/day data and provide positive reinforcement	One-on-one session with participant/parent and CanMOVE HCP	Motivation	Persuasion, incentivization	Prompting focus on past success Prompt rewards contingent on effort or progress toward behavior Prompt review of behavioral goals
Identify individual barriers to physical activity and discuss possible solutions		Motivation, opportunity	Persuasion, incentivization	Barrier identification and problem solving
Set a new steps per day goal based on prior results		Motivation	Persuasion, incentivization	Goal setting (behavior)
Create an action plan to achieve steps/day goal (see Table 3 for example action plan items)		Motivation, opportunity	Motivation, opportunity	Action planning
Provide support to carry out action plan	CanMOVE HCP allocated 1 h/week to assist implementation of action plan items	Motivation, opportunity	Motivation, opportunity	Action planning

***Phase 3***				
***Consolidation sessions 1–4***				
***(Repeat Theme 3 weekly)***				

BCT, behavior change technique; CALO-RE, Coventry, Aberdeen, and London – Refined; HCP; healthcare professional, HRQOL, health-related quality of life.

*Behavior Change Wheel intervention functions (50): Education, increasing knowledge or understanding; Persuasion, using communication to induce positive or negative feelings or stimulate action; Incentivization, creating expectation of reward; Coercion, creating an expectation of punishment or cost; Training, imparting skills; Restriction, using rules to reduce the opportunity to engage in the target reducing the behavior; Environmental restructuring, changing the physical or social context; Modeling, providing an example for people to aspire to or imitate; Enablement, increasing means/reducing barriers to increase capability or opportunity.

“Goal setting” and “self-monitoring” were identified as key strategies. Reduced motivation and self-efficacy are commonly reported barriers to physical activity (60). Giving children and adolescents the means to set goals and monitor progress in real time creates a sense of control that is rarely afforded in other aspects of their care (61). For delivery, activity monitors were selected. Activity monitors can also be used to apply a variety of behavior change techniques (62, 63). They are increasingly used within the pediatric settings (42, 64–67) and literature supports their use in motivating physical activity behavior, especially as part of a broader intervention plan (68). Using activity monitors to quantify physical activity via daily steps provides children/adolescents with a means to approximate the amount of physical activity they undertake in real-time. It is acknowledged that daily steps are one representation of physical activity, not taking account of other parameters such as intensity and frequency. However, daily steps are an accessible means by which to set and monitor physical activity goals (69). Rather than offering support that relies upon extrinsic motivation and staff supervision, activity monitors can facilitate intrinsic motivation through providing a means to self-manage behavior.

“Demonstration” was another key behavioral change strategy identified. For children and adolescents with cancer, an experience of physical impairment and reduced opportunity for activity has the potential to lead to a belief they are unable, or it is unsafe, to engage in physical activity. Through education and participating in an active demonstration session with a trained healthcare professional, opportunities for positive movement experiences can be identified. This builds confidence in a child/adolescent’s own ability to move.

“Planned social support,” “barrier identification and problem solving,” and “action planning” were also identified (70–73). Parental support is a key determinant of physical activity behavior in children and adolescents (74, 75). In the cancer treatment setting, negative perceptions toward physical activity can be reinforced by parents leading to perpetuating the sick role of the child/adolescent and a belief physical activity is unsafe (76). Parents can play a strong protective and advocacy role in the care of their child with cancer (77). In order to utilize this influential role, involvement of the family unit was identified as important. Facilitating opportunity for families to collaborate with their child/adolescent as a team gives control over how they engage with physical activity, enabling formulation of self-determined solutions specific to their interests and family context.

Through “environmental restructuring,” CanMOVE aims to encourage members of the medical multidisciplinary team to engage in a child/adolescent’s physical activity goals. The priorities that exist within an organization can impact a child/adolescent’s physical activity (78). Providing a means for other members of the treatment team to engage could result in additional motivation and opportunity for physical activity through facilitating changes in work practices and routines.

### The intervention: CanMOVE

**FIGURE 2:**
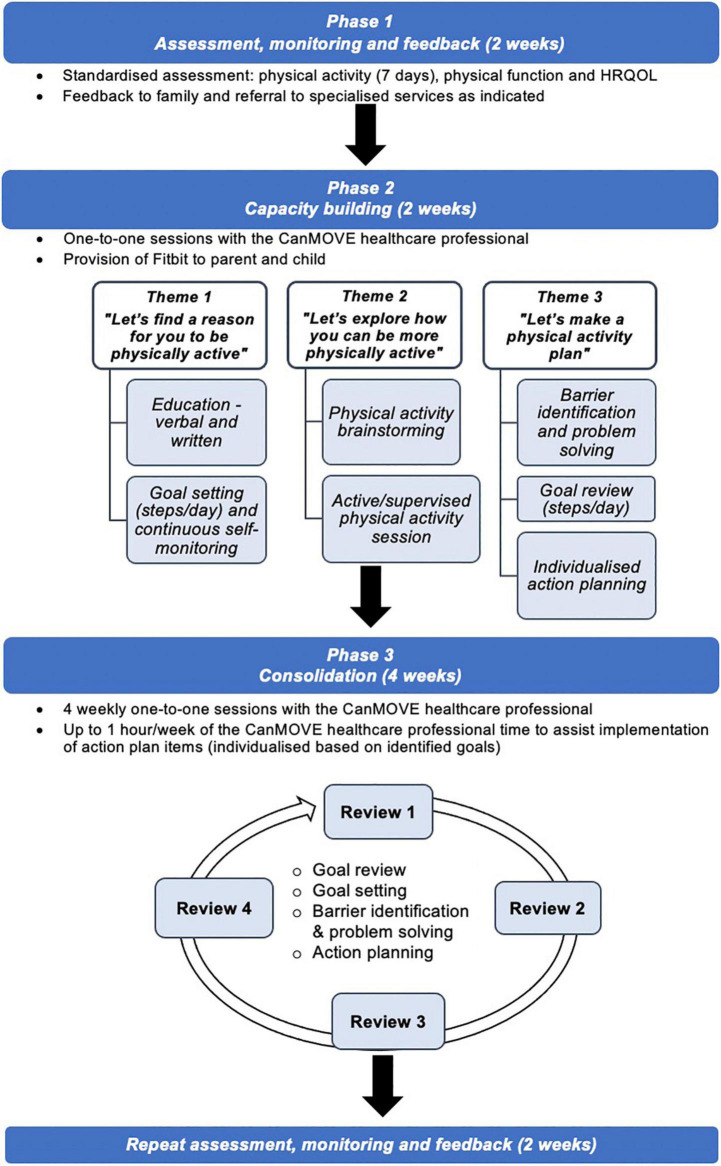
CanMOVE intervention model.

The name “CanMOVE” was selected to promote the idea that even in the context of acute cancer treatment, children and adolescents can be physically active. It is a flexible, individualized intervention tailored to suit the unique, and often changing, context of each child/adolescent. The intervention includes three phases run over 10 weeks: Assessment, Monitoring and Feedback (4 weeks), Capacity Building (2 weeks) and Consolidation (4 weeks) (Figure 2). The program is designed to be implemented by a healthcare professional, termed the “CanMOVE HCP.” This professional will have specific training in exercise and rehabilitation for children with cancer, such as an exercise physiologist or physiotherapist. The intent is for CanMOVE to run parallel to existing hospital or community-based therapy services. Where appropriate CanMOVE sessions can be conducted remotely to accommodate both the home and hospital environment, and overcome any isolation restrictions.

#### Phase 1: Assessment, monitoring, and feedback

This phase occurs at the beginning (2 weeks) and the end of the intervention (2 weeks). It includes objective assessment of physical activity, physical function (e.g., gross motor skills, cardiovascular function, functional tasks) and health-related quality of life (HRQOL). Each assessment outcome is discussed with the child/adolescent and their parent to build self-awareness of their current level of physical activity and learn about factors contributing to it (i.e., physical function and mental health). Assessing across two time points provides an opportunity to highlight and celebrate any improvements overtime. The assessment of physical function also provides opportunity to identify impairments requiring more intensive therapeutic input. In this phase, referrals can be made to additional services, for instance in the case of a vincristine neuropathy. There is insufficient evidence to support the selection of outcome measures to assess physical function in this population (79). Further psychometric analysis is required to inform the selection of assessment tools that may be utilized in this phase.

#### Phase 2: Capacity building

##### Theme 1: “Let’s find a reason for you to be physically active”

Theme 1 explores self-identified motivations toward physical activity. Education is individually tailored to identify motivating factors for them, and their parent/s. Here the CanMOVE HCP seeks to define the broad nature of physical activity, re-framing it as something that is achievable, fun, and part of the everyday routine. The benefits of physical activity are also discussed, specifically in the context of cancer treatment. A booklet specifically about physical activity and cancer treatment is provided (80). At the conclusion of the session, the child/adolescent is asked to identify 1–3 reasons why being physically active is important and beneficial for them.

The child/adolescent and one parent are provided with an activity monitor which is used to set an individualized daily step target. The daily steps target will act to broadly represent their participation in physical activity throughout the day. Together the child/adolescent and parent work toward their daily target. The initial daily step goal is formulated collaboratively taking into consideration results from the baseline assessment and current medical management. Progress toward their goal can be monitored continuously in real-time via the activity monitor. The daily step goal will be displayed in their hospital room (or at home) and communicated to the treating team via their medical record and multi-disciplinary team meetings.

##### Theme 2: “Let’s explore how you can be more physically active”

Theme 2 involves collaboratively brainstorming how the child/adolescent can be more physically active in their environment, whether that be at home or in the hospital setting. Within this session children will be encouraged to reflect upon what physical activity is, what they currently do, what they are able to do, and what they would like to do. In doing so, the child/adolescent is supported to identify new ways they can introduce physical activity opportunities into their daily routine. Identified strategies will aim to reflect the broad nature of ‘physical activity’ (25). For example, this may include activities of daily living, play, a structured exercise routine, sports skills, walking, and/or planned social interactions and hobbies that can incorporate incidental physical activity.

The child/adolescent will then participate in a physical activity session with the CanMOVE HCP. Activities completed will be tailored to the child/adolescent’s interests, treatment, abilities, and safety restrictions. Only activities the child/adolescent can carry out independently (or with the assistance of their parent) will be incorporated. If equipment, toys, technology, or active gaming are used, they must be readily available to the child/adolescent for independent use. This session aims to offer a positive movement experience that is fun and build confidence in their ability to move.

##### Theme 3: “Let’s make a physical activity plan”

Theme 3 aims to devise a physical activity plan in partnership with the child/adolescent and parent/s. Within this session, progress toward their daily step goal is reviewed. Positive reinforcement is provided in response to the child/adolescent making attempts to achieve the daily step goal. A list of barriers and facilitators to goal attainment are formulated. Factors that are within their realm of control are identified and potential solutions brainstormed. Here the daily step goal can be altered to make it more achievable or to motivate a challenge, a decision to be made in the context of upcoming treatment plans. An action plan will be formulated to work toward the daily steps target. Action plan items will comprise individualized strategies to assist in overcoming identified barriers. Tasks will be agreed upon and implemented by the child/adolescent and parent. The aim here is to support families to make independent choices regarding how the child/adolescent chooses to move, and motivate a shift toward a more physically active daily routine.

In addition, the CanMOVE HCP will allocate one hour to assist implementation of action plan items over the course of the following week. Action plan items will involve the broader treating medical and nursing team where able. An example of a barrier identification and action plan can be viewed in Table 3. In cases where psychological or physical impairments are identified and cannot be addressed adequately within the scope of the CanMOVE program, the CanMOVE HCP will collaborate with specialized therapy services and referrals made as indicated.

**TABLE 3:** Example barriers and action plan items.

Barriers	Potential action plan items	Carried out by
Admitted on hospital ward – unsure how to be physically active, nothing to do	Loan equipment/games/toys to encourage physical activity in line with interests Facilitate a plan for regular time outdoors/off ward with medical team Develop a plan to restrict screen time Plan social visits with friends and family	Child/adolescent, parent, CanMOVE HCP CanMOVE HCP/medical and nursing team Child/adolescent, parent Child/adolescent, parent
	Attend team meetings and facilitate opportunities to be physically active through existing services, i.e., play therapy Carry out additional supervised physical activity sessions to increase confidence and promote independence	CanMOVE HCP CanMOVE HCP
	Plan ADLs to participate in (etc. dressing, showering, making own snacks) Facilitate interaction with other children on the ward Develop and schedule independent exercise program based on needs and interests Start a physical activity routine, e.g., ADLs, walking, independent exercises, sport activities/games	Child/adolescent, parent CanMOVE HCP, nursing team, parent Child/adolescent, parent, CanMOVE HCP Child/adolescent, parent, CanMOVE HCP
Fatigue and nausea with current treatment	Plan rest time Education on pacing	Child/adolescent, parent, CanMOVE HCP Child/adolescent, parent, CanMOVE HCP
Tripping when walking	Discus with medical team need for physiotherapy referral for additional assessment and management	CanMOVE HCP, medical team, hospital physiotherapists
Lack of motivation to get up and move	Display prompts and cues to encourage physical activity Discuss goal with nursing staff and encourage their involvement in supporting their goal attainment	CanMOVE HCP CanMOVE HCP, medical and nursing team
Long periods of time on intravenous line	Schedule line-free time with nursing staff	CanMOVE HCP, medical and nursing team

ADLs, activities of daily living; HCP, healthcare professional.

#### Phase 3: Consolidation

Four “consolidation” sessions will be conducted to evaluate and modify intervention strategies based on their success in bringing about behavior change. Each week the daily steps data for the previous week will be discussed and a new goal set for the coming week. The daily step goal will aim to increase each week. However, to ensure goals are achievable, it may be maintained or decreased based on individual circumstances, such as upcoming hospital admissions/discharges, medical treatments and/or setbacks. Any new barriers and facilitators identified will be discussed. Success of action plan strategies will be reviewed, and items removed or added as indicated. An additional one hour of CanMOVE HCP time can be used to assist in carrying out action plan items each week.

### Outcomes

**FIGURE 3:**
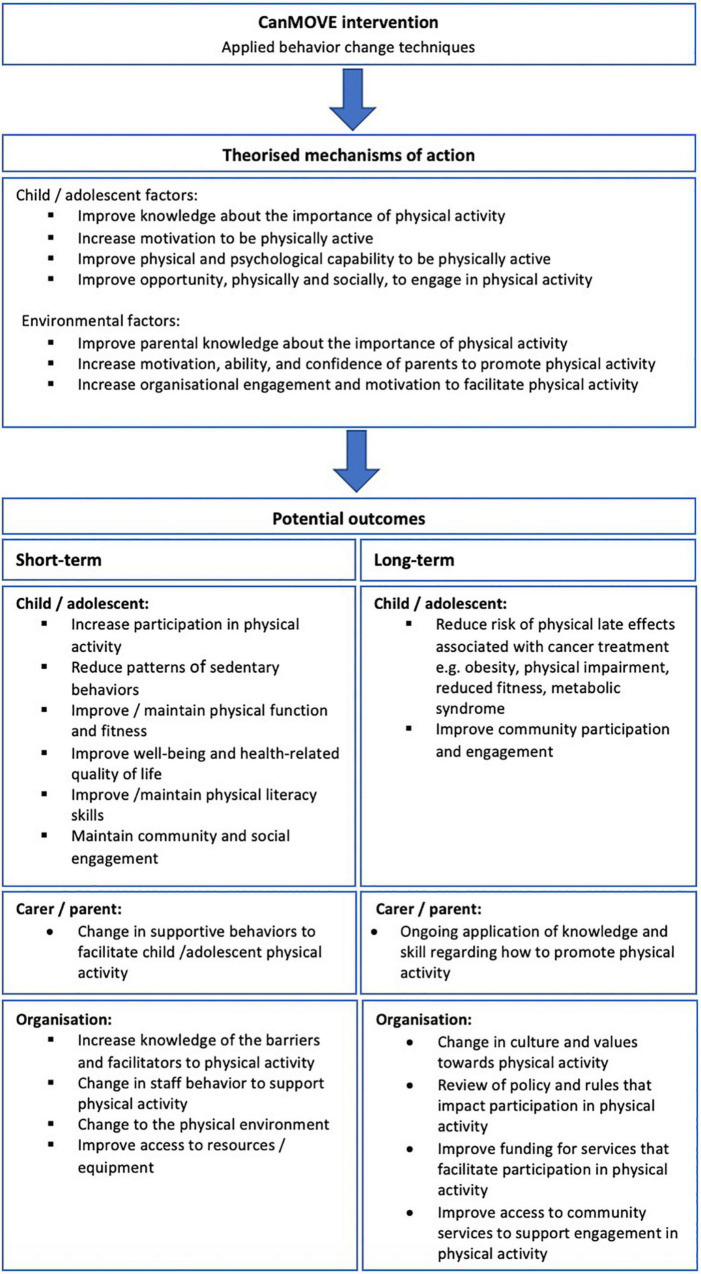
Outcomes.

The primary outcome of the CanMOVE intervention is to facilitate change in physical activity behavior in children undergoing acute cancer treatment. There may be additional potential benefits if CanMOVE is implemented in a clinical setting. The potential short- and long-term outcomes, along with their theorized mechanisms of action can be found in Figure 3. Prior to clinical implementation, CanMOVE must first be piloted for feasibility and undergo further development to ensure safety, acceptability, and optimum efficacy.

## Discussion

CanMOVE is a complex intervention that takes a novel and proactive approach to physical activity promotion. With a focus on behavior change, CanMOVE aims to promote positive movement experiences and maximize the family’s capacity toward physical activity. The design process was transparent, theory-driven and informed by qualitative data. The Behavior Change Wheel process necessitated a deep understanding of the target behavior, population and environment (50). Although time consuming, developing a clear behavioral diagnosis specific to the desired population ensured all subsequent design decisions were relevant to the population. The result is an intervention that targets specific physical activity challenges faced by children and adolescents within the acute cancer treatment setting. Intervention strategies identified for CanMOVE promote physical activity as necessary, enjoyable, and achievable in the acute cancer treatment setting. This perspective is in-line with recently released physical activity guidelines for children with cancer (81). Given the complex determinants of physical activity behavior for children with cancer, it is important to acknowledge that CanMOVE is only one element within a multi-system approach required to promote physical activity for this population.

CanMOVE seeks to complement, rather than replace specialized therapy services that provide physical assessment, intervention, and rehabilitation. Without the availability of therapists to address treatment related physical impairments (for example post-surgical impairments, myopathy and neuropathy), children and adolescents with compromised physical function will find it challenging to be physically active. CanMOVE incorporates a mechanism whereby physical function is monitored, with referrals made on a need basis. This approach ensures physical impairments are identified and treated promptly, while maximizing the efficiency of specialized service provision. Success, however, relies upon the selection of psychometrically robust outcome measures (79), and adequate services in place to provide additional therapy as needed. Given the protracted nature of acute cancer treatment, the provision of monitoring and follow up after the completion of CanMOVE is another consideration. The Stoplight program is an example of a clinical service that utilizes monitoring and targeted exercise provision with positive results (82).

Treating organizations have a role to play to ensure hospital environments, professional services and staff values are conducive to physical activity engagement. The social-ecological model provides a framework to describe the multiple levels of influence to be considered in working toward the promotion of positive health behaviors (83). In addition to addressing factors on an individual and interpersonal level, there is a need for change at the organizational level. CanMOVE invites treating teams to participate in a child/adolescent’s physical activity promotion, yet there are other positive changes an organization could make to support physical activity. These changes fall within the policy categories of the Behavior Change Wheel, such as environmental planning, service provision and a review of hospital guidelines (50). For example, often equipment and spaces that promote physical activity are not readily available to families. Altering treatment environments to allow space and independent access to equipment is a positive change that could promote physical activity. Other examples include education programs for nursing staff on physical activity promotion, including physical activity goals into medical treatment plans, and a review of hospital polies that restrict physical activity.

Feasibility evaluation is a vital step in the complex intervention design process (37). Prior to implementation, CanMOVE will be assessed for feasibility in a non-randomized pilot study (84) against criteria designed by Bowen et al. (85). The undertaking of a theoretical approach to intervention design will aid this evaluation. Without clearly defined “active ingredients” of the intervention, understanding what worked, and how, can be difficult to isolate. A comprehensive analysis of feasibility, utilizing qualitative and quantitative data (86), enables a deeper understanding of intervention elements such as: which were implemented successfully, which were effective, and the potential mechanisms underlying any observed changes in behavior. It also works to answer questions such as how well an intervention fits within a clinical setting and how acceptable it is. Addressing these questions is essential to inform future intervention development decisions and clinical implementation strategies. In depth analysis of the barriers and facilitators to physical activity reported by participants during the pilot study will also help the inform future intervention development decisions, and guide potential changes to the environment and services. Future design considerations for CanMOVE will include when to time the intervention, which outcome measures to use, how to engage the multi-disciplinary team, and how changes of behavior changes may be maintained over the entire length of acute treatment and into survivorship (87).

CanMOVE endeavors to promote positive physical activity experiences through maximizing a child/adolescent’s capacity, motivation and opportunities for movement. It aims to change how parents, children and adolescents think about physical activity. Results will ultimately inform the implementation of services within the pediatric cancer setting. This type of intervention, however, cannot stand alone. Meaningful change relies upon organizations providing specialized services and environments that promote and facilitate participation in physical activity. The theoretical design process underpinning the design of CanMOVE is an important stepping-stone toward understanding how to improve physical activity participation for children and adolescents in this setting. It also has potential application to other pediatric chronic health populations where physical activity participation is challenged in the hospital setting.

## Data availability statement

The original contributions presented in this study are included in the article, further inquiries can be directed to the corresponding author.

## Author contributions

All authors listed have made a substantial, direct, and intellectual contribution to the work, and approved it for publication.

## References

[1] Deisenroth A Söntgerath R Schuster AJ von Busch C Huber G Eckert K Muscle strength and quality of life in patients with childhood cancer at early phase of primary treatment. *Pediatr Hematol Oncol.* (2016) 33:393–407. 27690707 10.1080/08880018.2016.1219796

[2] Elmantaser M Stewart G Young D Duncan R Gibson B Ahmed S. Skeletal morbidity in children receiving chemotherapy for acute lymphoblastic leukaemia. *Arch Dis Child.* (2010) 95:805–9. 20576660 10.1136/adc.2009.172528

[3] Fuemmeler BF Pendzich MK Clark K Lovelady C Rosoff P Blatt J Diet, physical activity, and body composition changes during the first year of treatment for childhood acute leukemia and lymphoma. *J Pediatr Hematol Oncol.* (2013) 35:437–43. 10.1097/MPH.0b013e318279cd3e 23211695 PMC3606649

[4] Lavoie Smith EM Li L Chiang C Thomas K Hutchinson RJ Wells EM Patterns and severity of vincristine-induced peripheral neuropathy in children with acute lymphoblastic leukemia. *J Peripher Nerv Syst.* (2015) 20:37–46. 25977177 10.1111/jns.12114 PMC4610712

[5] Thorsteinsson T Larsen HB Schmiegelow K Thing LF Krustrup P Pedersen MT Cardiorespiratory fitness and physical function in children with cancer from diagnosis throughout treatment. *BMJ Open Sport Exerc Med.* (2017) 3:e000179. 10.1136/bmjsem-2016-000179 28761697 PMC5530132

[6] Ness KK Kaste SC Zhu L Pui CH Jeha S Nathan PC Skeletal, neuromuscular and fitness impairments among children with newly diagnosed acute lymphoblastic leukemia. *Leuk Lymphoma.* (2015) 56:1004–11. 10.3109/10428194.2014.944519 25030039 PMC4336225

[7] Bhakta N Liu Q Ness KK Baassiri M Eissa H Yeo F The cumulative burden of surviving childhood cancer: an initial report from the St Jude lifetime cohort study (SJLIFE). *Lancet.* (2017) 390:2569–82. 28890157 10.1016/S0140-6736(17)31610-0 PMC5798235

[8] Ness KK Leisenring WM Huang S Hudson MM Gurney JG Whelan K Predictors of inactive lifestyle among adult survivors of childhood cancer: a report from the childhood cancer survivor study. *Cancer.* (2009) 115:1984–94. 19224548 10.1002/cncr.24209 PMC2692052

[9] Smith WA Li C Nottage KA Mulrooney DA Armstrong GT Lanctot JQ Lifestyle and metabolic syndrome in adult survivors of childhood cancer: a report from the St. Jude lifetime cohort study. *Cancer.* (2014) 120:2742–50. 25070001 10.1002/cncr.28670 PMC4165406

[10] Baade PD Youlden DR Valery PC Hassall T Ward L Green AC Trends in incidence of childhood cancer in Australia, 1983–2006. *Br J Cancer.* (2010) 102:620–6. 10.1038/sj.bjc.6605503 20051948 PMC2822940

[11] Carson V Stone M Faulkner G. Patterns of sedentary behavior and weight status among children. *Pediatr Exerc Sci.* (2014) 26:95–102. 24092774 10.1123/pes.2013-0061

[12] Pellegrini AD Smith PK. Physical activity play: the nature and function of a neglected aspect of play. *Child Dev.* (1998) 69:577–98. 9680672

[13] Lam KK Li WH Chiu SY Chan GC. The impact of cancer and its treatment on physical activity levels and quality of life among young Hong Kong Chinese cancer patients. *Eur J Oncol Nurs.* (2016) 21:83–9. 10.1016/j.ejon.2016.01.007 26952682

[14] Winter C Müller C Brandes M Brinkmann A Hoffmann C Hardes J Level of activity in children undergoing cancer treatment. *Pediatr Blood Cancer.* (2009) 53:438–43. 19415742 10.1002/pbc.22055

[15] Hochhauser D Tobias JS. *Cancer and Its Management.* Somerset: John Wiley & Sons, Incorporated (2014).

[16] Stout NL Baima J Swisher AK Winters-Stone KM Welsh J. A systematic review of exercise systematic reviews in the cancer literature (2005-2017). *PM R.* (2017) 9:S347–84. 10.1016/j.pmrj.2017.07.074 28942909 PMC5679711

[17] Myers RM Balsamo L Lu X Devidas M Hunger SP Carroll WL A prospective study of anxiety, depression, and behavioral changes in the first year after a diagnosis of childhood acute lymphoblastic leukemia. *Cancer.* (2014) 120:1417–25. 10.1002/cncr.28578 24473774 PMC4319360

[18] White J Flohr JA Winter SS Vener J Feinauer LR Ransdell LB. Potential benefits of physical activity for children with acute lymphoblastic leukaemia. *Dev Neurorehabil.* (2005) 8:53–8. 10.1080/13638490410001727428 15799136

[19] Friedman DN Tonorezos ES Cohen P. Diabetes and metabolic syndrome in survivors of childhood cancer. *Horm Res Paediatr.* (2019) 91:118–27. 30650414 10.1159/000495698 PMC6610586

[20] Morales JS Valenzuela PL Rincón-Castanedo C Takken T Fiuza-Luces C Santos-Lozano A Exercise training in childhood cancer: a systematic review and meta-analysis of randomized controlled trials. *Cancer Treat Rev.* (2018) 70:154–67. 10.1016/j.ctrv.2018.08.012 30218787

[21] Klika R Tamburini A Galanti G Mascherini G Stefani L. The role of exercise in pediatric and adolescent cancers: a review of assessments and suggestions for clinical implementation. *J Funct Morphol Kinesiol.* (2018) 3:7.

[22] Baumann FT Bloch W Beulertz J. Clinical exercise interventions in pediatric oncology: a systematic review. *Pediatr Res.* (2013) 74:366–74. 23857296 10.1038/pr.2013.123

[23] Wurz A Daeggelmann J Albinati N Kronlund L Chamorro-Vina C Culos-Reed SN. Physical activity programs for children diagnosed with cancer: an international environmental scan. *Support Care Cancer.* (2019) 27:1153. 10.1007/s00520-019-04669-5 30726517

[24] Grimshaw SL Taylor NF Shields N. The feasibility of physical activity interventions during the intense treatment phase for children and adolescents with cancer: a systematic review. *Pediatr Blood Cancer.* (2016) 63:1586–93. 10.1002/pbc.26010 27186955

[25] Caspersen CJ Powell KE Christenson GM. Physical activity, exercise, and physical fitness: definitions and distinctions for health-related research. *Public Health Rep.* (1985) 100:126–31. 3920711 PMC1424733

[26] Corr AM Liu W Bishop M Pappo A Srivastava DK Neel M Feasibility and functional outcomes of children and adolescents undergoing preoperative chemotherapy prior to a limb-sparing procedure or amputation. *Rehabil Oncol.* (2017) 35:38–45. 28948112 PMC5609724

[27] Fiuza-Luces C Padilla JR Soares-Miranda L Santana-Sosa E Quiroga JV Santos-Lozano A Exercise intervention in pediatric patients with solid tumors: the physical activity in pediatric cancer trial. *Med Sci Sports Exerc.* (2017) 49:223–30. 27631396 10.1249/MSS.0000000000001094

[28] Gohar SF Comito M Price J Marchese V. Feasibility and parent satisfaction of a physical therapy intervention program for children with acute lymphoblastic leukemia in the first 6 months of medical treatment. *Pediatr Blood Cancer.* (2011) 56:799–804. 10.1002/pbc.22713 21370414

[29] Hartman A te Winkel ML van Beek RD de Muinck Keizer-Schrama SM Kemper HC Hop WC A randomized trial investigating an exercise program to prevent reduction of bone mineral density and impairment of motor performance during treatment for childhood acute lymphoblastic leukemia. *Pediatr Blood Cancer.* (2009) 53:64–71. 10.1002/pbc.21942 19283791

[30] Winter C Müller C Hardes J Gosheger G Boos J Rosenbaum D. The effect of individualized exercise interventions during treatment in pediatric patients with a malignant bone tumor. *Support Care Cancer.* (2013) 21:1629–36. 23292667 10.1007/s00520-012-1707-1

[31] Zucchetti G Rossi F Chamorro Vina C Bertorello N Fagioli F. Exercise program for children and adolescents with leukemia and lymphoma during treatment: a comprehensive review. *Pediatric Blood Cancer.* (2018) 65:e26924. 10.1002/pbc.26924 29314654

[32] Rustler V Hagerty M Daeggelmann J Marjerrison S Bloch W Baumann FT. Exercise interventions for patients with pediatric cancer during inpatient acute care: a systematic review of literature. *Pediatr Blood Cancer.* (2017) 64:e26567 10.1002/pbc.26567 28423225

[33] Braam KI van der Torre P Takken T Veening MA van Dulmen-den Broeder E Kaspers GJL. Physical exercise training interventions for children and young adults during and after treatment for childhood cancer. *Cochrane Libr.* (2013) 4:CD008796. 10.1002/14651858.CD008796.pub2 23633361

[34] Wurz A McLaughlin E Chamorro Viña C Grimshaw SL Hamari L Götte M Advancing the field of pediatric exercise oncology: research and innovation needs. *Curr Oncol.* (2021) 28:619–29. 10.3390/curroncol28010061 33498499 PMC7924382

[35] Brown MC Sharp L Sniehotta FF Skinner R Araújo-Soares V. The development of health behaviour change interventions for childhood cancer survivors: the need for a behavioural science approach. *Pediatr Blood Cancer.* (2020) 67:e28500. 10.1002/pbc.28500 32614142

[36] Cross A Howlett N Sheffield D. Social ecological interventions to increase physical activity in children and young people living with and beyond cancer: a systematic review. *Psychol Health.* (2020) 35:1477–96. 10.1080/08870446.2020.1759601 32468857

[37] Craig P Dieppe P Macintyre S Michie S Nazareth I Petticrew M. Developing and evaluating complex interventions: the new medical research council guidance. *BMJ.* (2008) 337:a1655. 10.1136/bmj.a1655 18824488 PMC2769032

[38] Buchan DS Ollis S Thomas NE Baker JS. Physical activity behaviour: an overview of current and emergent theoretical practices. *J Obes.* (2012) 2012:546459. 10.1155/2012/546459 22778918 PMC3388376

[39] Imms C. Children with cerebral palsy participate: a review of the literature. *Disabil Rehabil.* (2008) 30:1867–84. 19037780 10.1080/09638280701673542

[40] Ferreira I Van Der Horst K Wendel-Vos W Kremers S Van Lenthe FJ Brug J. Environmental correlates of physical activity in youth – a review and update. *Obes Rev.* (2007) 8:129–54. 17300279 10.1111/j.1467-789X.2006.00264.x

[41] Cox CL Zhu L Kaste SC Srivastava K Barnes L Nathan PC Modifying bone mineral density, physical function, and quality of life in children with acute lymphoblastic leukemia. *Pediatr Blood Cancer.* (2017) 65:e26929. 10.1002/pbc.26929 29286560 PMC5821547

[42] Götte M Kesting SV Gerss J Rosenbaum D Boos J. Feasibility and effects of a home-based intervention using activity trackers on achievement of individual goals, quality of life and motor performance in patients with paediatric cancer. *BMJ Open Sport Exerc Med.* (2018) 4:e000322. 10.1136/bmjsem-2017-000322 29765699 PMC5950644

[43] Lam KKW Li WHC Chung OK Ho KY Chiu SY Lam HS An integrated experiential training programme with coaching to promote physical activity, and reduce fatigue among children with cancer: a randomised controlled trial. *Patient Educ Couns.* (2018) 101:1947–56. 10.1016/j.pec.2018.07.008 30007765

[44] Nielsen MKF Christensen JF Frandsen TL Thorsteinsson T Andersen LB Christensen KB Effects of a physical activity program from diagnosis on cardiorespiratory fitness in children with cancer: a national non-randomized controlled trial. *BMC Med.* (2020) 18:175. 10.1186/s12916-020-01634-6 32624004 PMC7336676

[45] Skivington K Matthews L Simpson S Craig P Baird J Blazeby J A new framework for developing and evaluating complex interventions: update of medical research council guidance. *BMJ.* (2021) 374:n2061. 10.1136/bmj.n2061 34593508 PMC8482308

[46] O’Cathain A Croot L Duncan E Rousseau N Sworn K Turner KM Guidance on how to develop complex interventions to improve health and healthcare. *BMJ Open.* (2019) 9:e029954. 10.1136/bmjopen-2019-029954 31420394 PMC6701588

[47] Michie S Abraham C. Interventions to change health behaviours: evidence-based or evidence-inspired? *Psychol Health.* (2004) 19:29–49.

[48] Michie S Johnston M Francis J Hardeman W Eccles M. From theory to intervention: mapping theoretically derived behavioural determinants to behaviour change techniques. *Appl Psychol.* (2008) 57:660–80.

[49] Willmott TJ Pang B Rundle-Thiele S. Capability, opportunity, and motivation: an across contexts empirical examination of the COM-B model. *BMC Public Health.* (2021) 21:1014. 10.1186/s12889-021-11019-w 34051788 PMC8164288

[50] Michie S Van Stralen MM West R. The behaviour change wheel: a new method for characterising and designing behaviour change interventions. *Implement Sci.* (2011) 6:1–12. 21513547 10.1186/1748-5908-6-42 PMC3096582

[51] Kinnear FJ Wainwright E Bourne JE Lithander FE Hamilton-Shield J Searle A. The development of a theory informed behaviour change intervention to improve adherence to dietary and physical activity treatment guidelines in individuals with familial hypercholesterolaemia (FH). *BMC Health Serv Res.* (2020) 20:27. 10.1186/s12913-019-4869-4 31914998 PMC6950899

[52] Martin R Murtagh EM. An intervention to improve the physical activity levels of children: design and rationale of the ‘active classrooms’ cluster randomised controlled trial. *Contemp Clin Trials.* (2015) 41:180–91. 10.1016/j.cct.2015.01.019 25657052

[53] Murtagh E Barnes A McMullen J Morgan P. Mothers and teenage daughters walking to health: using the behaviour change wheel to develop an intervention to improve adolescent girls’ physical activity. *Public Health.* (2018) 158:37–46. 10.1016/j.puhe.2018.01.012 29544174

[54] Webb J Foster J Poulter E. Increasing the frequency of physical activity very brief advice for cancer patients. Development of an intervention using the behaviour change wheel. *Public Health.* (2016) 133:45–56. 10.1016/j.puhe.2015.12.009 26822162

[55] Grimshaw SL Taylor NF Mechinaud F Conyers R Shields N. Physical activity for children undergoing acute cancer treatment: a qualitative study of parental perspectives. *Pediatr Blood Cancer.* (2020) 67:e28264. 10.1002/pbc.28264 32277806

[56] Lam KK Ho Cheung William L Ho KY Chung OK Chan CF. Factors contributing to the low physical activity level for Hong Kong Chinese children hospitalised with cancer: an exploratory study. *J Clin Nurs.* (2017) 26:190–201. 10.1111/jocn.13495 27487435

[57] Gotte M Kesting S Winter C Rosenbaum D Boos J. Experience of barriers and motivations for physical activities and exercise during treatment of pediatric patients with cancer. *Pediatr Blood Cancer.* (2014) 61:1632–7. 24753116 10.1002/pbc.25071

[58] Thorsteinsson T Schmiegelow K Thing LF Andersen LB Helms AS Ingersgaard MV Classmates motivate childhood cancer patients to participate in physical activity during treatment: a qualitative study. *Eur J Cancer Care (Engl).* (2019) 28:e13121. 10.1111/ecc.13121 31215079

[59] Michie S Ashford S Sniehotta FF Dombrowski SU Bishop A French DP. A refined taxonomy of behaviour change techniques to help people change their physical activity and healthy eating behaviours: the CALO-RE taxonomy. *Psychol Health.* (2011) 26:1479–98. 10.1080/08870446.2010.540664 21678185

[60] Bar-Mor G Bar-Tal Y Krulik T Zeevi B. Self-efficacy and physical activity in adolescents with trivial, mild, or moderate congenital cardiac malformations. *Cardiol Young.* (2000) 10:561–6. 10.1017/S1047951100008829 11117387

[61] Gibbins J Steinhardt K Beinart H. A systematic review of qualitative studies exploring the experience of parents whose child is diagnosed and treated for cancer. *J Pediatr Oncol Nurs.* (2012) 29:253–71. 10.1177/1043454212452791 22907681

[62] Lyons EJ Lewis ZH Mayrsohn BG Rowland JL. Behavior change techniques implemented in electronic lifestyle activity monitors: a systematic content analysis. *J Med Internet Res.* (2014) 16:e192. 10.2196/jmir.3469 25131661 PMC4147713

[63] Mercer K Li M Giangregorio L Burns C Grindrod K. Behavior change techniques present in wearable activity trackers: a critical analysis. *JMIR Mhealth Uhealth.* (2016) 4:e40. 10.2196/mhealth.4461 27122452 PMC4917727

[64] Le A Mitchell HR Zheng DJ Rotatori J Fahey JT Ness KK A home-based physical activity intervention using activity trackers in survivors of childhood cancer: a pilot study. *Pediatr Blood Cancer.* (2017) 64:387–94. 10.1002/pbc.26235 27615711

[65] Hooke MC Gilchrist L Tanner L Hart N Withycombe JS. Use of a fitness tracker to promote physical activity in children with acute lymphoblastic leukemia. *Pediatr Blood Cancer.* (2016) 63:684–9. 10.1002/pbc.25860 26756736

[66] Mendoza JA Baker KS Moreno MA Whitlock K Abbey-Lambertz M Waite A A fitbit and facebook mHealth intervention for promoting physical activity among adolescent and young adult childhood cancer survivors: a pilot study. *Pediatr Blood Cancer.* (2017) 64:e26660. 10.1002/pbc.26660 28618158

[67] Schofield L Mummery WK Schofield G. Effects of a controlled pedometer-intervention trial for low-active adolescent girls. *Med Sci Sports Exerc.* (2005) 37:1414–20. 10.1249/01.mss.0000174889.89600.e3 16118591

[68] Sullivan AN Lachman ME. Behavior change with fitness technology in sedentary adults: a review of the evidence for increasing physical activity. *Front Public Health.* (2017) 4:289. 10.3389/fpubh.2016.00289 28123997 PMC5225122

[69] Kraus WE Janz KF Powell KE Campbell WW Jakicic JM Troiano RP Daily Step counts for measuring physical activity exposure and its relation to health. *Med Sci Sports Exerc.* (2019) 51:1206–12. 10.1249/MSS.0000000000001932 31095077 PMC6527133

[70] Michie S Abraham C Whittington C McAteer J Gupta S. Effective techniques in healthy eating and physical activity interventions: a meta-regression. *Health Psychol.* (2009) 28:690–701. 10.1037/a0016136 19916637

[71] Greaves CJ Sheppard KE Abraham C Hardeman W Roden M Evans PH Systematic review of reviews of intervention components associated with increased effectiveness in dietary and physical activity interventions. *BMC Public Health.* (2011) 11:119. 10.1186/1471-2458-11-119 21333011 PMC3048531

[72] Kahn EB Ramsey LT Brownson RC Heath GW Howze EH Powell KE The effectiveness of interventions to increase physical activity. A systematic review. *Am J Prev Med.* (2002) 22:73–107. 10.1016/S0749-3797(02)00434-8 11985936

[73] Rhodes RE Pfaeffli LA. Mediators of physical activity behaviour change among adult non-clinical populations: a review update. *Int J Behav Nutr Phys Act.* (2010) 7:1–11. 10.1186/1479-5868-7-37 20459781 PMC2876989

[74] Trost SG Loprinzi PD. Parental influences on physical activity behavior in children and adolescents: a brief review. *Am J Lifestyle Med.* (2011) 5:171–81. 10.1177/1559827610387236

[75] Golan M. Parents as agents of change in childhood obesity – from research to practice. *Int J Pediatr Obes.* (2006) 1:66–76. 10.1080/17477160600644272 17907317

[76] Le A Li F Mitchell HR Kadan-Lottick N. Exercise practices, preferences, and barriers in the pediatric oncology population. *Pediatr Blood Cancer.* (2015) 62:2177–84. 10.1186/s12913-016-1423-5 26207515

[77] Kars MC Duijnstee MSH Pool A Van Delden JJM Grypdonck MHF. Being there: parenting the child with acute lymphoblastic leukaemia. *J Clin Nurs.* (2008) 17:1553–62. 10.1111/j.1365-2702.2007.02235.x 18482117

[78] Keats MR Culos-Reed SN Courneya KS. An examination of the beliefs, attitudes and counselling practices of paediatric oncologists toward physical activity: a provincial survey. *Paediatr Child Health.* (2007) 12:289–93. 10.1093/pch/12.4.289 19030372 PMC2528686

[79] Grimshaw SL Taylor NF Mechinaud F Shields N. Assessment of physical function in children with cancer: a systematic review. *Pediatr Blood Cancer.* (2018) 65:e27369. 10.1002/pbc.27369 30094937

[80] Service PIC. *Paediatric Integrated Cancer Service. Family Resources, Everyday Care, Physical Activity Booklet.* (2021) Available online at: https://ed492f82-48ec-4169-8a4b-3c5f0d1cdad4.filesusr.com/ugd/3fb492_01d1ce6feefc4e63af5725f64fb3899d.pdf

[81] Wurz A McLaughlin E Lategan C Chamorro Viña C Grimshaw SL Hamari L The international pediatric oncology exercise guidelines (iPOEG). *Transl Behav Med.* (2021) 11:1915–22. 10.1093/tbm/ibab028 34037786 PMC8604278

[82] Tanner LR Hooke MC. Improving body function and minimizing activity limitations in pediatric leukemia survivors: the lasting impact of the stoplight program. *Pediatr Blood Cancer.* (2019) 66:e27596. 10.1002/pbc.27596 30609245

[83] McLeroy KR Bibeau D Steckler A Glanz K. An ecological perspective on health promotion programs. *Health Educ Q.* (1988) 15:351–77. 10.1177/109019818801500401 3068205

[84] Eldridge SM Lancaster GA Campbell MJ Thabane L Hopewell S Coleman CL Defining feasibility and pilot studies in preparation for randomised controlled trials: development of a conceptual framework. *PLoS One.* (2016) 11:e0150205. 10.1371/journal.pone.0150205 26978655 PMC4792418

[85] Bowen DJ Kreuter M Spring B Cofta-Woerpel L Linnan L Weiner D How we design feasibility studies. *Am J Prev Med.* (2009) 36:452–7. 10.1016/j.amepre.2009.02.002 19362699 PMC2859314

[86] Moore GF Audrey S Barker M Bond L Bonell C Hardeman W Process evaluation of complex interventions: medical research council guidance. *BMJ.* (2015) 350:h1258. 10.1136/bmj.h1258 25791983 PMC4366184

[87] Grimmett C Foster C Bradbury K Lally P May CR Myall M Exploring maintenance of physical activity behaviour change among people living with and beyond gastrointestinal cancer: a cross-sectional qualitative study and typology. *BMJ Open.* (2020) 10:e037136. 10.1136/bmjopen-2020-037136 33122311 PMC7597473

